# Dementia-Related Anxiety and Its Impact on Social Connectedness and Suicide Risk

**DOI:** 10.1093/geroni/igaf122.457

**Published:** 2025-12-31

**Authors:** Molly Maxfield, Allie Peckham, Dara James, Laura Lathrop, Amy Fiske

**Affiliations:** Arizona State University, Phoenix, Arizona, United States; Arizona State University, Phoenix, Arizona, United States; Arizona State University, Tempe, Arizona, United States; University of Denver, Denver, Colorado, United States; West Virginia University, Morgantown, West Virginia, United States

## Abstract

Alzheimer’s disease and related dementias (ADRD) lead to declines in independence, financial and psychological burden to those diagnosed and close others, and decreased social engagement, all of which can contribute to dementia-related anxiety (DRA). DRA involves anxiety about currently or eventually having dementia, often driven by self-perceived ADRD risk, feared changes to the self and identity, and anticipated interpersonal consequences. The interpersonal theory of suicide suggests that fundamental human desires to belong, contribute, and be needed are critical to well-being, and when undermined, the feelings of exclusion and burdensomeness is so painful, suicidal ideation (SI) may result (Van Orden et al., 2010). Therefore, to the extent that DRA has an interpersonal component, it is likely associated with SI. In a mixed methods study of 50 midlife and older adults without a dementia diagnosis (Mage = 70.80, SDage = 6.02), 42.0% of the sample anticipated suicidal ideation (14.0%) or death ideation (DI; 28.0%) if they were diagnosed with dementia. Three themes emerged from statements of anticipated SI and DI: fear of becoming a burden to others, devaluation of life/loss of self with dementia, and the desire for (and anticipated thwarting of) personal control. Since most DRA research focuses on cognitively healthy participants, it is unclear whether anticipated SI and DI translates to increased suicide risk post-diagnosis. Given evidence that death by suicide is highest within 90 days of ADRD diagnosis (Schmutte et al., 2021), we examine the intersection of three late-life public health challenges: ADRD, social disconnectedness, and suicide risk.

